# Impact of extracorporeal haemoadsorption during prolonged cardiopulmonary bypass on the incidence of acute kidney injury

**DOI:** 10.1051/ject/2024004

**Published:** 2024-06-18

**Authors:** Nilufar Jabayeva, Bolat Bekishev, Timur Lesbekov, Zhuldyz Nurmykhametova, Rymbay Kaliyev, Linar Faizov, Aidyn Kuanyshbek, Robertas Samalavicius

**Affiliations:** 1 Department of Anesthesiology and Intensive Care, National Research Cardiac Surgery Center Astana 010000 Kazakhstan; 2 Department of Adult Cardiac Surgery, National Research Cardiac Surgery Center Astana 010000 Kazakhstan; 3 Department of Perfusiology and assisted circulation laboratory, National Research Cardiac Surgery Center Astana 010000 Kazakhstan; 4 Department of Anesthesia, Intensive Care and Pain Management, Vilnius University Hospital Santariskiu Clinics Vilnius 01100 Lithuania

**Keywords:** Prolonged cardiopulmonary bypass, Haemoadsorption, Systemic inflammatory response syndrome, Acute kidney injury

## Abstract

The usage of cardiopulmonary bypass (CPB) in cardiothoracic surgery contributes to the activation of the inflammatory response. In certain cases, the systemic inflammatory response may be immoderate, leading to organ dysfunction, such as acute renal failure or multiorgan dysfunction. This study aimed to examine the effect of haemoadsorption (HA) therapy on inflammatory markers and renal damage indices during cardiopulmonary bypass and in the early postoperative period. We conducted a retrospective analysis of prospectively collected data in a single tertiary care center on patients operated between January 2021 and May 2022. The levels of inflammatory markers and renal parameters in blood samples (Interleukin (IL) 6, C-reactive protein (CRP), white blood cells, lactate, procalcitonin (PCT), and NT-proBNP, urea, creatinine, glomerular filtration rate (GFR), mechanical ventilation days and intensive care unit (ICU) days) were compared between the three groups. Data from the Jafron HA 330 (*n* = 20) and CytoSorb300 (*n* = 20) groups were compared with those from the control group (*n* = 20). All patients underwent cardiopulmonary bypass for more than 120 min. Baseline patient characteristics were similar in all three groups. Acute kidney injury (AKI) was diagnosed in 17 patients (28.3%); seven patients were in the Jafron HA 330, two in the CytoSorb300, and eight in the control group. We found that IL1α, IL 6, IL8, Lactate dehydrogenase, PCT, NT-proBNP, CRP, Leukocyte, and TNFα had no significant or clinical difference between the CytoSorb 300 and Jafron HA 330 adsorber groups. Our results indicate that haemoadsorption therapy does not significantly reduce the risk of AKI after prolonged CPB, but decreases the need for renal replacement therapy.

## Introduction

Cardiopulmonary bypass (CPB) takes over the function of the heart and lungs during open-heart surgery, with a bloodless field and immobile heart [1, 2]. However, CPB is often associated with systemic inflammatory responses due to traumatic stress, monocyte/macrophage activation, and coagulation [3, 4]. During CPB, the extrication of several inflammatory molecules (C3a, C5a, histamine, IL-6, IL-8, and TNF-α) causes activation of cellular responses, which leads to the development of systemic inflammation, increased vascular pressure, permeability, and thrombosis [5].

It has been demonstrated that the effects of CPB-induced systemic inflammation can lead to adverse outcomes such as hemodynamic instability, coagulopathy, acute renal and other organ failure, and even death [6–10]. Extracorporeal removal of inflammatory cytokines may improve the prognosis of patients following CPB use, given the association between elevated levels of pro-inflammatory cytokines and adverse clinical outcomes [11, 12]. Different approaches have been created to reduce systemic inflammatory responses during CPB, such as pharmaceutical and non-medicament approaches (removing inflammatory molecules by hemoperfusion, reducing surface area using mini circuits, and improving the biocompatibility of extracorporeal surfaces) [3, 13]. However, Clive Landis et al. [3] reported that only about a third of these approaches showed clinically improved efficacy. Therefore, the development of a new strategy to reduce systemic inflammation caused by CPB is still relevant.

The duration and severity of the harmful triggers associated with CPB might have a negative impact on the recovery of the patient after the surgery. In fact, the systemic inflammatory background is exacerbated by massive therapeutic invasion, surgical trauma, CPB [6], and blood product transfusion [14]. Heart surgery and CPB invariably cause systemic inflammatory response syndrome. Release of many cytokines, including IL-1, IL-6, IL-8, IL 10, complement C3/C4, and tumor necrosis factor-α PCT, Leucocytes are characteristic of the inflammation and contribute to postoperative acute kidney injury (AKI) [6].

For the diagnosis of cardiac surgery-associated acute kidney injury (CSA-AKI) the Kidney Disease Improving Global Outcomes (KDIGO) classification has become a consensus with greater sensitivity in the detection of AKI postoperatively than other classifications [6].

We conducted this study to compare levels of inflammatory markers and the incidence of CSA-AKI in the early postoperative period after prolonged CPB using CytoSorb-300 and HA-330.

## Materials and methods

We conducted a retrospective review of prospectively collected data in a single tertiary care center between January 2021 and May 2022. The study was approved by the Local Bioethics Committee (No. 01-74/2021 from 10/06/20), and registered in ClinicalTrials.gov PRS, Protocol registration and results system (NCT05090930). Two types of HA devices were used – Jafron HA 330 (HA 330, Jafron Biomedical Co., Ltd. China) and CytoSorb 300 (CytoSorb^®^, cartridge, Cytosorbents Europe GmbH, Germany) (see Supplementary table for characteristics of the adsorbent cartridges). Sixty patients with a CPB (LivaNova Sorin Stockert S5, Germany, tubing pack, and oxygenator Inspire8 with an integrated filter Livanova, Affinity Fusion Medtronic, Alone Eurosets) duration exceeding 120 min. formed the study group. The mean age of the patients was 53 (±14) years in the HA 330 group vs. 49 (±18) years in the CytoSorb 300 group and 51 (±12) years in the control group, with 45%, 55%, and 65% of patients being male respectively. The mean body mass index (BMI) and Apache II score were similar in all groups. The baseline parameters and surgery characteristics are presented in Table 1.

**Table 1:** Patients characteristics.

Characteristics	HA 330 group (*n* = 20)	CytoSorb 300 group (*n* = 20)	Control group (*n* = 20)	P value
Demography				
Mean age (years)	53.19 ± 14.44	49.15 ± 18.15	51.68 ± 12.58	0.26
Male, *n* (%)	9 (45%)	11 (55%)	13 (65%)	
Female, *n* (%)	11 (55%)	9 (45%)	7 (35%)	
Comorbidity, mean ± SD				
BMI	26.58 ± 4.46	26.94 ± 3.7	28.18 ± 5.8	0.14
Apache II score, points	16.73 ± 2.55	14.32 ± 6.25	12.73 ± 8.29	0.10
Diabetes requiring insulin	2 (10%)	1 (5%)	1 (5%)	
Ischemic stroke	1 (5%)	1 (5%)	1 (5%)	
Cardiac surgery				
Heart valve surgery, *n* (%)	10 (50%)	11 (55%)	9 (45%)	
GABG, *n* (%)	1 (5%)	1 (5%)	3 (15%)	
Surgery on aorta, *n* (%)	7 (35%)	4 (20%)	7 (35%)	
Heart transplantation	2 (10%)	1 (5%)	0	
LVAD	0	3 (15%)	1 (5%)	
Reoperation	6 (30%)	2 (10%)	2 (10%)	
Emergency surgery	1 (5%)	3 (15%)	2 (10%)	
Cardiogenic shock	1 (5%)	1 (5%)	0	
CPB time (min)	218.14 ± 86.92	201.85 ± 65.39	194.45 ± 42.42	0.5
Cross clamp time (min)	121.23 ± 66.91	103.25 ± 56.86	115 ± 44.69	0.5
Circulatory arrest (min)	4.04 ± 8.08	8.45 ± 9.47	3.5 ± 8.31	0.14

At the time of analysis for this article, 40 patients had cytokine adsorption and 20 patients without adsorption while on prolonged CPB scheduled for elective complex cardiac surgery with CPB duration >120 min. All patients were intraoperatively single randomized by 1:1:1 into three study groups: Cytosorb, CS (*n* = 20; installed in the CPB circuit), Jafron HA, JHA (*n* = 20; installed in the CPB circuit) and Control, CO (*n* = 20, usual care, neither Cytosorb nor Jafron during CPB). The primary outcomes of the study were the postoperative level of inflammatory markers (IL-1, 6, 8; CRP, Leukocyte, Lactate, PCT, NT-proBNP, TNF-α), and incidence of acute kidney injury by KDIGO classification. Secondary outcomes of interest were duration of mechanical ventilation, length of ICU and hospital stay, and hospital mortality. Each patient received three consecutive HA procedures, (the first procedure intraoperatively during CPB, and two consecutive procedures in the postoperative period).

Preparatory washing of the adsorbents and heparinization during the procedures was carried out according to the manufacturer’s instructions. Anticoagulation was achieved by administering heparin (individual dosage, according to the laboratory data and the condition of the post-operative bleeding). All adsorption procedures were performed in a standard manner in HA 330 and Cytosorb groups. The first HA procedure was initiated intraoperatively from the start of the CPB. The second HA procedure was conducted in the early postoperative period in the ICU 6 h after surgery. The third HA procedure was conducted consecutively after the end of the second procedure. In all cases, the cartridges were incorporated into a Continuous Veno-Venous Hemofiltration (CVVH) machine or haemoperfusion machine (Jafron Company) if there was no need for renal replacement therapy (RRT). The only difference was the manufacturer-defined duration of adsorption: 6 h for HA 330, and 24 h for Cytosorb.

**Figure 1:**
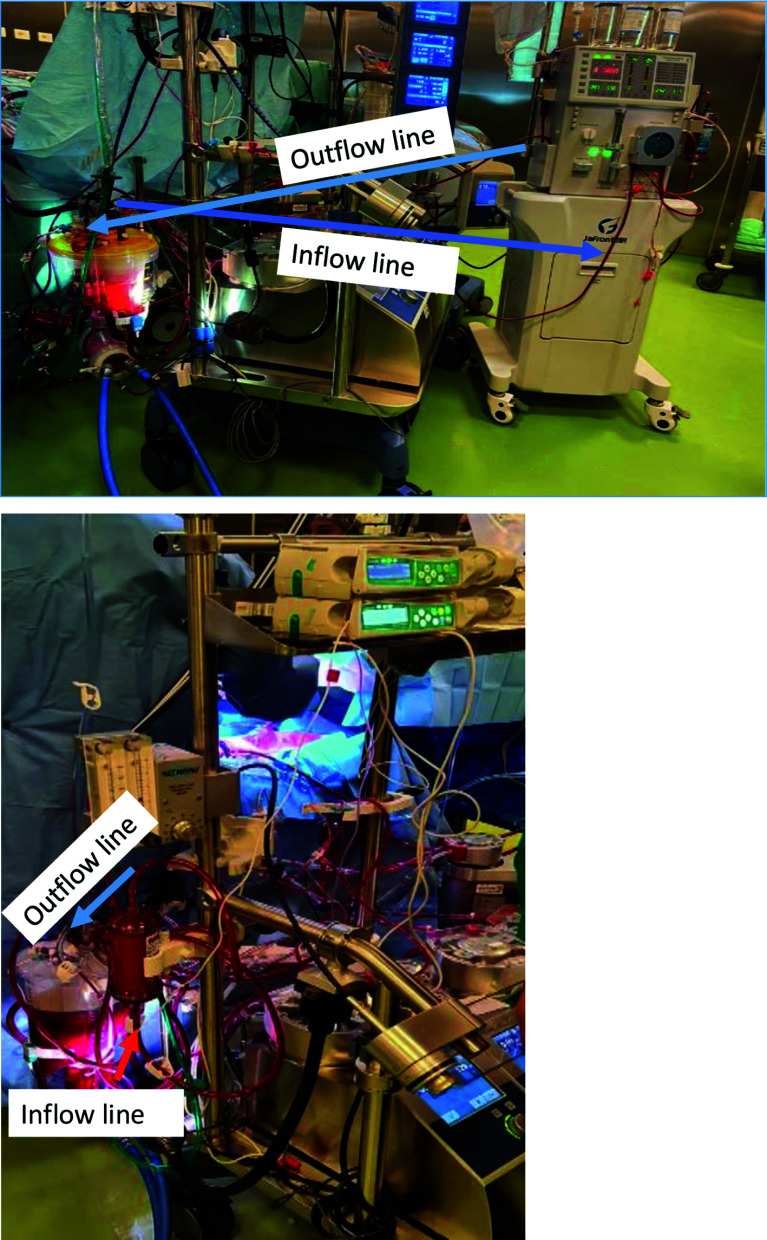
Picture of extracorporeal blood purification and CPB.

Intraoperative HA was performed using a standard CPB roller pump (Stockert S5, LivaNova Deutschland GmbH) with a mean arterial pressure of 68–84 mmHg and temperature control (35 °C). The HA 330 device was placed in the CPB venous circuit (Figure 1), using a haemoperfusion machine. The blood flow rate on the haemoperfusion machine was 160–200 ml/min. The Cytosorb cartridge was placed in the CPB without a haemoperfusion machine with an inflow arterial line and returned to the venous line.

The duration of HA with the CytoSorb 300 cartridge was 24 h and with the HA 330 cartridge – 6 h according to the manufacturer’s recommendations.

### Data collection

Trends of inflammatory markers (IL-6, IL-8, IL-10, TNF-α, CRP, Leukocytes, Lactate, PCT, NT-proBNP, urea, creatinine) were assessed. Blood samples were collected seven times: the – first before surgery, the – second 1 hour on CPB, the third – at the end of CPB, the fourth – 2 h after surgery, the fifth – 6 h after surgery, the sixth – 12 h after surgery and the seventh – 24 h after surgery.

The APACHE II score was used to assess patients 6 h after ICU admission following surgery.

### Ethical statement

The study was approved by the Local Bioethics Committee of the National Research Cardiac Surgery Center (No. 01-74/2021 from 10/06/20), and registered in ClinicalTrials.gov Protocol and Outcome Registration System (NCT05042622). All patients’ personal information was coded and data were anonymized at the time of data collection to protect patients’ rights and not to divulge their personal information. The researchers received an electronic database with only patient demographic and clinical information, which was analyzed and presented only in aggregate form, further guaranteeing data confidentiality.

### Statistical analysis

Statistical analyses were performed using SPSS (version 26 IBM, SPSS Inc., Chicago, IL, USA). Demographic and clinical baseline data are presented as mean and standard deviation, expressed through minimum and maximum, for metric variables or absolute frequencies for categorical variables. Differences between groups were analyzed by using the analysis of variance (ANOVA) test for to compare the means of two or more independent samples. A significant difference was assumed for *p*-values less than 0.05. Results are presented as medians with interquartile ranges.

## Results

Intraoperative parameters were comparable between the groups. The mean CPB time was 218 (±86) min in the HA 330 group versus 201 (±65) min in the CytoSorb 300 group, and 194 (±42) min in the control group.

The mean duration of postoperative mechanical ventilation was 3.33 ± 5.24 days versus 3.42 ± 5.5 days versus 7.7 ± 17.39 days in HA330, Cytosorb, and control group respectively. The duration of stay in the ICU (3.8 ± 7.24 vs. 4.47 ± 5.58 vs. 10 ± 17.52 days, respectively) was not significantly shorter in the HA330 group (*p* = 0.08).

Mortality was observed in all groups. There was one death in the HA-330 group, one death in the CytoSorb-300 group, and two deaths in the control group (Table 2).

**Table 2:** Secondary outcomes.

Mean ± standard deviation	HA 330 group (*n* = 20)	CytoSorb300 group (*n* = 20)	Control group (*n* = 20)	P value
ICU stay (days)	3.80 ± 7.24	4.47 ± 5.58	10.05 ± 17.52	0.08
Mechanical ventilation (days)	3.33 ± 5.24	3.42 ± 5.51	7.7 ± 17.39	0.17
Hospital stay (days)	24.05 ± 12.24	23.31 ± 14.38	28.63 ± 18.39	0.47
Mortality	1 (5%)	1 (5%)	2 (10%)	

**Figure 2:**
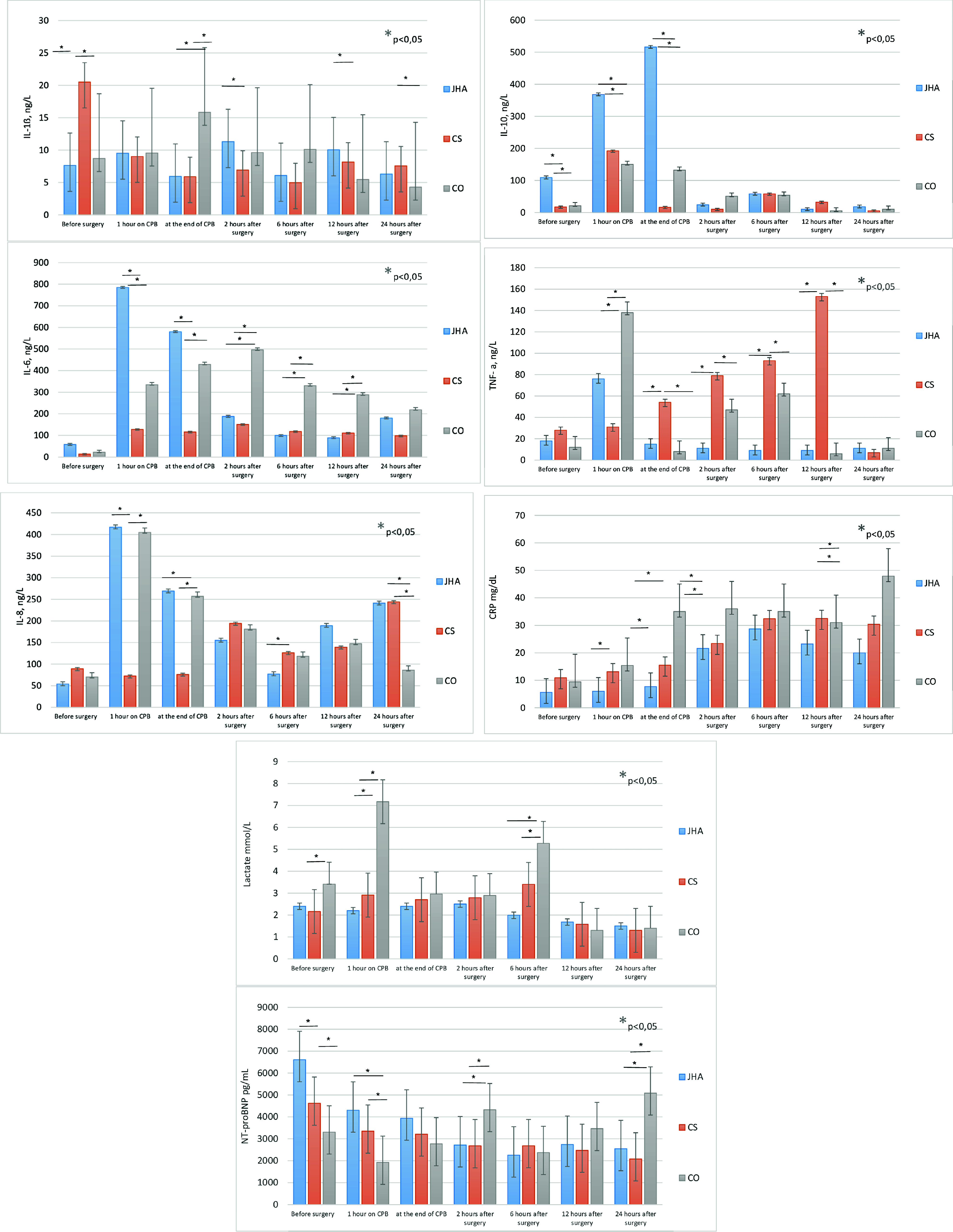
Cytokines and secondary outcome measures (a, b, c, d).

**Figure 3:**
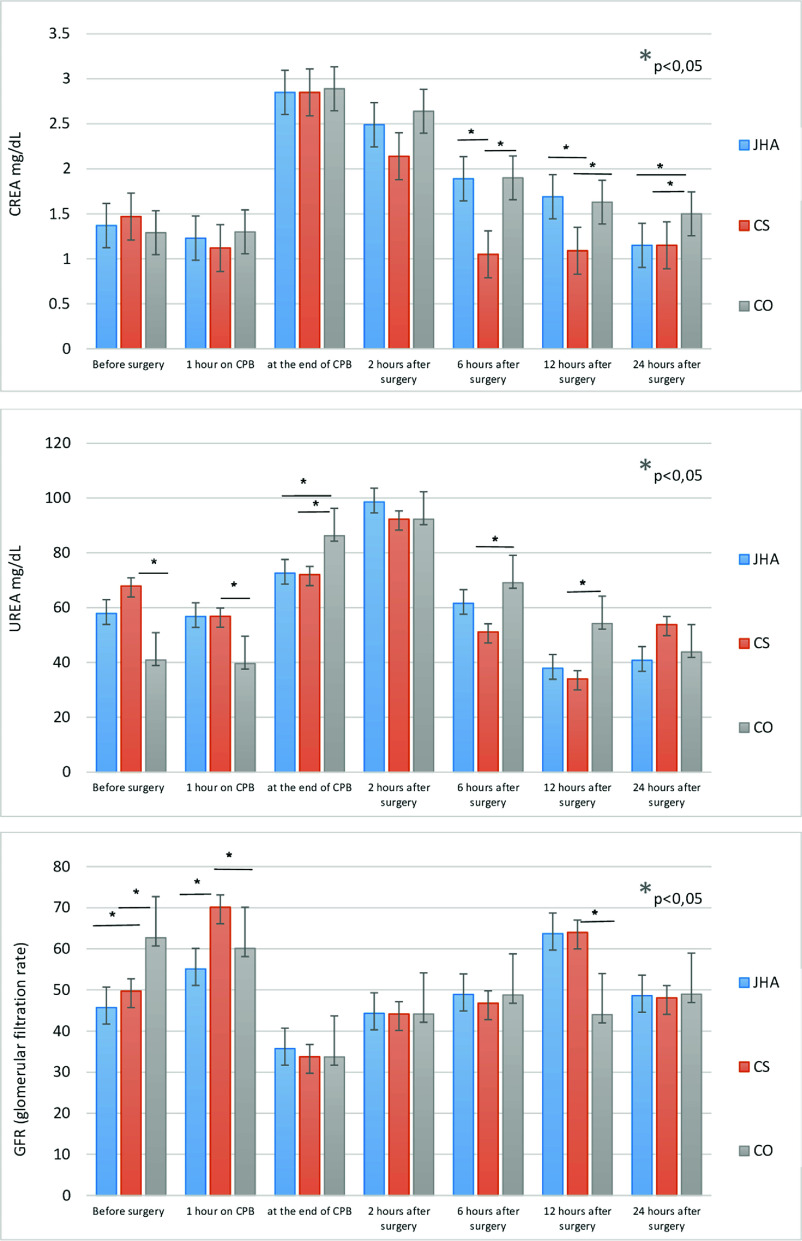
Data of kidney functions.

All patients had elevated inflammatory markers in the perioperative and postoperative periods. After 24 h of intensive therapy, inflammatory markers in the blood tended to decrease in all three groups (Figure 2). The laboratory data of HA330, CytoSorb 300, and control groups are shown in Figures 2 and 3. In our study, we found that the use of blood purification adsorbers during and after surgery with cardiopulmonary bypass was associated with a reduction of IL-6, CRP, and leucocytes. Patients in both HA groups demonstrated some different values of inflammatory markers throughout the first three days. For HA 330 there was a gradual decrease of IL 6, IL 8, and IL 10 after peak on the first day. Cytosorb cartridge showed a more prominent and stable decrease of IL 1 β, IL 6, and IL 8 throw-out implementation period of adsorption. Preoperative NT Pro-BNP levels were higher in the HA group. This could be explained by advanced heart failure in patients with multivalvular disease, redo surgery, and shock conditions that consisted HA group. After surgery, Pro-BNP levels increased more prominent in the control group indicating the possible influence of sorbents.

In the postoperative period, acute kidney injury was diagnosed in 17 patients (28.3%). Of these, seven patients were in the Jafron HA 330, two in the CytoSorb300, and eight in the control group. Severe early ischemic liver injury after cardiac surgery occurred in one patient in the Jafron HA 330 group, one in the CytoSorb300 group, and three patients in the control. Cardiac or respiratory failure requiring support with V-A ECMO was necessary in five patients in the Jafron HA 330 group, four in the CytoSorb-300 group, and in two patients in the control group. Increased pericardial bleeding as per Kirklin criteria of more than 500 ml during the first hours was found in the Jafron HA 330 group in three patients, as well as one in each of the CytoSorb 300 and control groups. Heparin-induced thrombocytopenia was laboratory and clinically diagnosed by one patient in the HA 330 and control groups. One patient in the group receiving CytoSorb300 had an ischemic stroke, see Table 3. Extracorporeal blood purification procedures in groups was shown on Table 4.

**Table 3:** Complications.

	HA330 group *n* = 20	CytoSorb-300 group *n* = 20	Control group *n* = 20	*P* value
Acute kidney injury, *n* (%)	7 (35%)	2 (10%)	8 (40%)	0.63
Renal replacement therapy quantity, *n*	28	30	67	0.05
Liver injury, *n* (%)	1 (5%)	1 (5%)	3 (15%)	0.41
Bleeding*, *n* (%)	3 (15%)	1 (5%)	1 (5%)	0.41
VA ECMO*, *n* (%)	5 (25%)	4 (20%)	2 (10%)	0.45
Heparin induced thrombocytopenia, *n* (%)	1 (5%)	0	1 (5%)	0.59

* Active bleeding in immediate postoperative period, more than 500 ml in 3 h. VA ECMO: Veno-arterial extracorporeal membrane oxygenation.

**Table 4:** Extracorporeal blood purification procedures.

	Quantity of procedures		
Type of procedure	HA330 group *n* = 20	CytoSorb-300 group (*n* = 20)	Control group (*n* = 20)
CVVHF, mean (range)	1	0	4
CVVHDF, mean (range)	5	4	5
CVVHD, mean (range)	1	0	6
OL-HDF, mean (range)	17	16	23
OL-HF, mean (range)	1	8	6
Intermittent HD, mean (range)	0	0	16
SLED, mean (range)	1	0	1
HP (HA 280), mean (range)	2	0	0
HP (HA330II), mean (range)	0	2	6
Total number of procedures	28	30	67

CVVHF – Continuous Veno-Venous Hemofiltration; CVVHDF – Continuous venovenous hemodiafiltration; CVVHD – Continuous venovenous hemodialysis; OL-HDF – Online haemodiafiltration; OL-HF – Online hemofiltration; Intermittent HD – Intermittent hemodialysis; SLED – sustained low-efficiency dialysis; HP (HA 280) – hemoperfusion cartridge HA 280; HP (HA330II) – hemoperfusion cartridge HA 330 II.

## Discussion

Excessively high levels of inflammatory mediators are common during critical illness and might be associated with adverse outcomes. The extracorporeal blood purification technologies with different types of adsorbents have been used and evaluated in various critical diseases [15–19], having been shown to be safe and effective, significantly improving hemodynamics and reducing the catecholamine requirement. Data for the use of this technology in patients undergoing cardiac surgery with cardio-pulmonary bypass remains scarce.

In a series of 16 cardiac surgery patients with systemic inflammatory response syndrome after CPB and following AKI, Träger et al. [20] reported that haemosorption treatment can decrease elevated cytokine levels (IL-6 and IL-8), stabilize impaired hemodynamics, and improve organ function. In a further study of 39 cardiac surgery patients with acute infective endocarditis who underwent valve replacement, Träger et al. [21] also demonstrated that haemosorption therapy may reduce the postoperative response of pivotal cytokines level, clinical parameters, and the requirement for vasopressors, improving hemodynamic stability and functional improvement of organ. Likewise, Nemeth et al. [22] reported that intraoperative hemoperfusion treatment was associated with a decreased need for vasopressors in patients undergoing heart transplantation. In our study, we found that the use of blood purification adsorbers during and after surgery with cardiopulmonary bypass was associated with a reduction of IL-6, CRP, and leucocytes. CRP levels showed lower levels through three days in the HA 330 group.

Studies have shown that a longer duration of cardiopulmonary bypass has a negative impact on the time of recovery following surgery with CPB [23, 24]. Many factors might be involved in delayed recovery following cardiopulmonary bypass, though the exact mechanism is unknown. In our series half of the patients had advanced valvular disease, one-third had aortic pathology and one-quarter were reoperated. The influence of HA should be evaluated in these subgroups separately in larger studies as they may have more patient-related risk factors for end organ failure. In addition, we evaluated only patients with long duration of cardiopulmonary bypass, hoping that the extracorporeal blood purification therapy might be of utmost importance in reducing the negative impact of cardiopulmonary bypass in these patients. Duration of CPB was found to be an independent predictor of acute renal failure [24]. However, in the present study, there was no statistically significant difference in the rate of postoperative renal failure in patients when blood purification was used. This might be due to the sample size and heterogenicity of patients.

The difference in NT-proBNP and TNF-α in the three groups can not be predicted. This effect should be studied as a cartridge saturation in the next studies.

Series HA cartridges are extensively used in China, and only some cases of fever and transient thrombocytopenia have been demonstrated [18, 20]. In our study, just one case of thrombocytopenia was detected in the Jafron HA group. Such results indicate that the sorbents are biocompatible and safe for haemosorption during CPB in cardiac surgery. Based on our data, the use of the HA procedure is possible and safe with both CytoSorb 300 and HA 330 adsorbents. The application of both adsorbers does not present any technical difficulties.

In summary, our results show that HA reduced intraoperative levels of inflammatory mediators, but had no effect on CSA-AKI in patients after long-term CPB. The heterogeneous nosological characteristics of patients and their small quantitative component, on the one hand, can serve as such an explanation. On the other hand, the standardized characteristics of the perfusion period may need to be personalized for the patient/nosology or reevaluated in terms of pumps used – roller or centrifugal.

For many years, the hemofiltration technique used during CPB did not demonstrate significant benefits in reducing inflammatory mediators. Currently used sorbents have a different mechanism of action, saturating the cartridge with inflammatory molecules (reducing their concentration in the bloodstream). So, the resulting blood load with inflammatory mediators at the intra- and early postperfusion period may facilitate early restoration of kidney function and less need for RRT.

### Study limitations

Our study has several limitations that need to be addressed. This is a single-center experience of the use of adsorbers during cardiac surgery with prolonged cardiopulmonary bypass. In addition, the number of patients included in the study limited the strength and generalization of the results.

## Conclusion

We found no significant difference in the incidence of CSA-AKI in the early postoperative period after long-term CPB using CytoSorb-300 and HA-330. HA therapy leads to lower levels of IL-6, CRP, and leucocytes. However, the differences in cytokine levels between patients who received haemosorption and control were not significant and the effect was not long-lasting. HA therapy did not significantly reduce the risk of CSA-AKI after long-term CPB, but it more than halved the need for substitution therapy.

## Data Availability

The data presented in this study is available upon request from the corresponding author.

## Supplementary material

The supplementary material of this article is available at https://ject.edpsciences.org/10.1051/ject/2024004/olm *Supplementary Table*: Characteristics of the CytoSorb300 and HA 330 cartridges.
