# Supplementary material for: Impact of extracorporeal haemoadsorption during prolonged cardiopulmonary bypass on the incidence of acute kidney injury
Source: J Extra Corpor Technol. 2024 Jun 18;56(2):45–54. doi: 10.1051/ject/2024004 (PMC11185140; doi:10.1051/ject/2024004)
Supplement: Supplementary file 1 — Supplementary Table: Characteristics of the CytoSorb300 and HA 330 cartridges. [file ject-56-45-s1.pdf]

Supplementary Table. Characteristics of the CytoSorb300 and HA 330 cartridges.

Type of cartridges	***CytoSorb 300 (Cytosorbents Europe GmbH).***	***HA 330(Jafron Biomedical Co., Ltd. China).***
Adsorbent:	Proprietary and patented cross-linked divinylbenzene polymer	a styrene-divinylbenzene copolymer
Cartridge Volume (ml)	300	330
Adsorption Spectrum:	Small and mid-size hydrophobic molecules up to a size of approximately 60kDa	With an approximate pore size distribution corresponding to a molecular weight range of 10–60 kDa
Maximum pressure limit:	760 mm Hg	750 mm Hg
Maximum procedure duration:	24 hours	6 hours
Anticoagulation:	heparin or citrate	heparin or citrate
Priming fluid, procedure and duration:	CytoSorb does not require priming/coating with heparin. Flushing with 2000 ml of 0.9% NaCL solution.<br>Priming takes approximately 5 minutes.	Flushing with 2500 mlof 0.9% NaCl solution+ 12500ME heparin.<br>The flushing takes about 50 minutes.
